# Trends and Risk Factors for Venous Thromboembolism Among Hospitalized Medical Patients

**DOI:** 10.1001/jamanetworkopen.2022.40373

**Published:** 2022-11-21

**Authors:** Elad Neeman, Vincent Liu, Pranita Mishra, Khanh K. Thai, James Xu, Heather A. Clancy, David Schlessinger, Raymond Liu

**Affiliations:** 1 Department of Hematology and Oncology, The Permanente Medical Group, San Rafael, California; 2 Division of Research, Kaiser Permanente Northern California, Oakland; 3 Internal Medicine Residency Program, Kaiser Permanente Northern California, San Francisco; 4 Department of Hematology and Oncology, The Permanente Medical Group, San Francisco, California

## Abstract

**Importance:**

While hospital-associated venous thromboembolism (HA-VTE) is a known complication of hospitalization, contemporary incidence and outcomes data are scarce and methodologically contested.

**Objective:**

To define and validate an automated electronic health record (EHR)–based algorithm for retrospective detection of HA-VTE and examine contemporary HA-VTE incidence, previously reported risk factors, and outcomes.

**Design, Setting, and Participants:**

This cohort study was conducted using hospital admissions between January 1, 2013, and June 30, 2021, with follow-up until December 31, 2021. All medical (non–intensive care unit) admissions at an integrated health care delivery system with 21 hospitals in Northern California during the study period were included. Data were analyzed from January to June 2022.

**Exposures:**

Previously reported risk factors associated with HA-VTE and administration of pharmacological prophylaxis were evaluated as factors associated with HA-VTE.

**Main Outcomes and Measures:**

Yearly incidence rates and timing of HA-VTE events overall and by subtype (deep vein thrombosis, pulmonary embolism, both, or unknown), as well as readmissions and mortality rates.

**Results:**

Among 1 112 014 hospitalizations involving 529 492 patients (268 797 [50.8%] women; 75 238 Asian [14.2%], 52 697 Black [10.0%], 79 398 Hispanic [15.0%], and 307 439 non-Hispanic White [58.1%]; median [IQR] age, 67.0 [54.0-79.0] years), there were 13 843 HA-VTE events (1.2% of admissions) occurring in 10 410 patients (2.0%). HA-VTE events increased from 307 of 29 095 hospitalizations (1.1%) in the first quarter of 2013 to 551 of 33 729 hospitalizations (1.6%) in the first quarter of 2021. Among all HA-VTE events, 10 746 events (77.6%) were first noted after discharge. In multivariable analyses, several factors were associated with increased odds of HA-VTE, including active cancer (adjusted odds ratio [aOR], 1.96; 95% CI, 1.85-2.08), prior VTE (aOR, 1.71; 95% CI, 1.63-1.79), and reduced mobility (aOR, 1.63; 95% CI, 1.50-1.77). Factors associated with decreased likelihood of HA-VTE included Asian race (vs non-Hispanic White: aOR, 0.65; 95% CI, 0.61-0.69), current admission for suspected stroke (aOR, 0.73; 95% CI, 0.65-0.81), and Hispanic ethnicity (vs non-Hispanic White: aOR, 0.81; 95% CI, 0.77-0.86). HA-VTE events were associated with increased risk of readmission (hazard ratio [HR], 3.33; 95% CI, 3.25-3.41) and mortality (HR, 1.63; 95% CI, 1.57-1.70).

**Conclusions and Relevance:**

This study found that HA-VTE events occurred in 1.2% of medical admissions, increased over time, and were associated with increased adverse outcomes. These findings suggest that approaches designed to mitigate occurrence and outcomes associated with HA-VTE may remain needed.

## Key Points

**Question:**

What are the risk factors, incidence, setting, and clinical outcomes of patients diagnosed with hospital-associated venous thromboembolism (HA-VTE)?

**Findings:**

In this cohort study of 1 112 014 medical (non–intensive care unit) admissions between January 2013 and June 2021, there were 13 843 HA-VTE events (1.2%), with increasing incidence over time. Most events (77.6%) happened after discharge, and HA-VTE was associated with increased risk for mortality and readmissions.

**Meaning:**

This study found that HA-VTE identified during or after a hospital admission was associated with adverse outcomes. This finding suggests that research relevant to prevention may be warranted.

## Teaser

This cohort study examines factors associated with hospital-associated venous thromboembolism and its incidence among hospitalized patients.

## Introduction

Hospital-associated venous thromboembolism (HA-VTE), commonly defined as deep vein thrombosis (DVT), pulmonary embolism (PE), or both occurring during or within 90 days of hospital admission, is a frequent complication of hospitalization, accounting for approximately one-half to two-thirds of VTE incidence worldwide.^1,2,3,4^ HA-VTE events are associated with substantial burdens.^5^ They are a leading factor associated with hospital mortality^6,7,8^ and lost disability-adjusted life-years.^5,9^ They are also associated with increased hospital length of stay,^7^ cost,^10^ long-term morbidity,^2^ and risk of recurrent thromboses.^11^ HA-VTE are considered highly preventable,^12^ and accordingly, professional societies recommend performing individualized HA-VTE risk assessments and considering prophylaxis for each admitted patient.^13,14^ Additionally, regulatory agencies consider HA-VTE measures in assessing hospital quality.^15^

Despite the association of HA-VTE with adverse patient outcomes, there is a paucity of reliable contemporary clinical data^7^ to guide current practice. This gap arises from several challenges: (1) lack of national surveillance of HA-VTE in the United States^7,16^; (2) limitations of existing epidemiological studies relating to their size^7^; (3) reliance on diagnosis codes and administrative data, which have been repeatedly reported to be inadequate in capturing HA-VTE^15,16,17,18,19^; (4) difficulty distinguishing between HA-VTE and VTE events that were present on admission, between acute VTE and history of VTE, and between confirmed vs suspected VTE^16,20^; (5) sole reliance on hospital discharge data^18^ despite evidence that most HA-VTE events were diagnosed after discharge^16^; and (6) potential lack of access to data on postdischarge events if they occurred in other health care systems or settings.^15^

We used detailed electronic health record (EHR) data within a highly integrated health care system to identify and characterize HA-VTE events in medical patients. We report HA-VTE incidence, trends, and demographic attributes, and previously reported risk factors among patients with HA-VTE and their clinical outcomes.

## Methods

This cohort study was approved by the Kaiser Permanente Northern California (KPNC) Institutional Review Board, which waived the requirement for informed consent because this research could not practicably be carried out without the waiver. This manuscript follows the Strengthening the Reporting of Observational Studies in Epidemiology (STROBE) reporting guideline for cohort studies.

KPNC is a highly integrated health care delivery system serving more than 4.5 million members, encompassing urban, suburban, and semirural areas, with a membership that constitutes more than 30% of the population in counties where the system is present, reflecting the diversity of these communities.^21^ Since 2008, KPNC has used an Epic-based EHR for member health records. All data for this study were extracted from the EHR and associated KPNC data systems.

### Hospitalization Cohort

We conducted a retrospective cohort study of all medical hospitalizations (not including direct intensive care unit admissions) among adult patients (aged ≥18 years) at any of 21 KPNC hospitals between January 1, 2013, and June 30, 2021. Medical hospitalizations were identified based on a standardized hospitalist admission order set used across hospitals throughout the study period. Hospitalizations outside of KPNC were not included unless the patient was eventually repatriated to a KPNC hospital.

### Hospital-Acquired Venous Thromboembolism (HA-VTE) Definition

We defined HA-VTE as a VTE event diagnosed at least 48 hours after admission and within 90 days after discharge (eFigure 1 in the Supplement). We assumed that VTE events diagnosed in the first 48 hours of admission were present on admission rather than acquired in the hospital.^16,22,23^ We also excluded HA-VTE events among hospitalizations in which any therapeutic dose of anticoagulation was used in the first 48 hours of admission given that we could not definitively exclude VTE events already present on admission (eAppendix in the Supplement).

To optimize the accuracy of our HA-VTE definition, we used multiple criteria to establish an HA-VTE event, adapting methods described in prior studies.^16,22^ We identified an HA-VTE event if there was a definite finding of PE on a computed tomography (CT) scan based on inclusion of specific tags in the CT report (eAppendix in the Supplement) or if an admission was associated with 1 or more indications of VTE diagnosis (new *International Classification of Diseases, Ninth Revision *[*ICD-9*] or *International Statistical Classification of Diseases and Related Health Problems, Tenth Revision *[*ICD-10*] codes for VTE, an “abnormal” vascular ultrasound, or a CT scan suspicious for PE) and 1 or more indications of VTE treatment, which could include the following: (1) first encounter with or new referral to the anticoagulation clinic for VTE, (2) new filled therapeutic anticoagulation prescription, (3) new *ICD-9* or *ICD-10* diagnosis code for long-term anticoagulation, (4) placement of a new inferior vena cava filter, or (5) in-hospital death during the index admission after starting therapeutic anticoagulation (see eFigure 2 in the Supplement for a graphical representation of HA-VTE criteria logic, eTables 1 and 2 in the Supplement for prevalence of specific HA-VTE criteria, and the eAppendix in the Supplement for detailed specifications). We further categorized HA-VTE events as DVT (ie, abnormal vascular ultrasound, new *ICD-9* or *ICD-10* code for DVT, or both), PE (ie, CT scan definitive or suspicious for PE, new *ICD-9* or *ICD-10* code for PE, or both), DVT and PE, or unknown VTE type if none of these subclassifications applied (eAppendix in the Supplement). The timing and setting (ie, inpatient vs outpatient) of each HA-VTE event were defined as the time and setting of the first criteria met for the diagnosis or treatment of HA-VTE.

We conducted random retrospective EHR reviews by hematology and internal medicine physicians (E.N., J.X., and R.L.) with iterative corrections to HA-VTE criteria until each review exhibited an accuracy of 90% or higher.^22^ Once criteria were finalized, we manually reviewed a random sample of 200 EHRs of HA-VTE events based on the algorithm and found that the approach yielded a 95% true positive rate (190 of 200 events [95.0%]). Because no standardized registry for confirmed HA-VTE events existed and because HA-VTE events were uncommon, we also explored false-negative rates and missed cases by reviewing a random sample of 100 admissions in which 2 or more HA-VTE criteria were met but were insufficient to meet HA-VTE logic definitions (eg, multiple indications of VTE treatment without an indication of diagnosis). This review exhibited a 95% true-negative rate (95 of 100 events [95.0%]).

The unit of analysis in our study was a single hospitalization; thus, patients could contribute more than 1 admission to the data set.^20^ When an HA-VTE event could have been attributed to more than 1 hospitalization, it was counted once and attributed to the most recent admission prior to the event. The times of hospital admission and discharge were established as the times that the corresponding admit and discharge EHR orders were signed.

We also analyzed HA-VTE risk factors at the individual patient level. Because most included HA-VTE risk factors were dynamic (eg, age, active cancer, and previous VTE), we randomly selected 1 admission per patient within the study period and included risk factor variable values as they were at the time of the selected admission.

We assessed demographic variables (ie, age, legal sex, race, ethnicity, and requirement for language interpreter) as they were at the time of index admission. Race and ethnicity were self reported and were assessed because they are known or suspected to be associated with HA-VTE rates. If a patient identified as Hispanic ethnicity, we categorized that individual’s race or ethnicity as Hispanic. Otherwise, ethnicity was defined as non-Hispanic. Race categories included Asian, Black, Native American, White, multiracial, unknown, and other. Multiracial, unknown, and other race categories were defined as unknown or other. Additional outcome data (ie, readmissions within 30 days of hospital discharge and mortality within 180 days from HA-VTE diagnosis) were gathered from EHRs.

### Patient Characteristics and Risk Factors Associated With HA-VTE

We defined 23 potential risk factors associated with HA-VTE based on prior studies, which were adapted to be used with retrospective EHR data. These included the 11 elements of the Padua Predictive Score,^24^ as well as 12 additional previously identified risk factors^25,26,27,28,29,30,31,32,33,34,35,36,37,38^: active cancer, previous VTE, bed rest or immobilization, thrombophilia, recent trauma or surgery, age 70 years or older, current admission for heart or respiratory failure, current admission for ischemic stroke, acute infection, body mass index (calculated as weight in kilograms divided by height in meters squared) of 30 or more, ongoing hormonal treatment, being an active tobacco smoker, poorly controlled diabetes (ie, hemoglobin A_1c_ ≥10.0% [to convert to proportion of total hemoglobin, multiply by 0.01]), low hemoglobin level (ie, <10.0 g/dL [to convert to grams per liter, multiply by 10.0]), high platelet count (ie, ≥500 ×10^3^/uL [to convert to ×10^9^ per liter, multiply by 1.0]), presence of an infusion port or a peripherally inserted central (PICC) line, recent use of a serotonergic antidepressant, microalbuminuria, high illness severity index scores^39^ (Comorbidity Point Score [COPS] ≥50 and Laboratory Acute Physiology Score [LAPS] ≥100) at time of admission, recent major hemorrhage (within 30 days prior to admission), race and ethnicity, requirement for language interpreter, and male sex. In addition, a covariate of pharmacological VTE prophylaxis in the first 48 hours after admission was defined based on completed medication orders (eAppendix in the Supplement).^3^

We conducted retrospective manual EHR review for each risk factor with iterative corrections made until we achieved an accuracy of 90% or higher.^22^ In addition, for Padua Predictive Score elements,^24^ we prospectively validated each element in a sample of 75 consecutive admissions in October and November 2021 based on manual EHR review by internal medicine residents (including J.X.); this exhibited 90% or greater accuracy (ie, no more than 7of 75 incongruent admissions) for each individual criterion and for the overall score.

### Statistical Analysis

Data were reported as mean (SD), median (IQR), or number (percentage). All reported descriptive statistic results have been rounded to the nearest single decimal place, and inferential and comparative statistic results (eg, hazard ratios [HRs]) were rounded to the nearest 2 decimal places. We assessed HA-VTE incidence quarterly over the study period and used Kaplan-Meier survival curves to display time-to-event data for HA-VTE timing, readmissions, and mortality. Log-rank test was used to assess differences between survival curves. A Cox proportional-hazard regression model was used to estimate HRs for mortality and readmission among HA-VTE subgroups. We used independent, 2-tailed, paired *t* tests, Pearson χ^2^ tests, and Wilcoxon rank sum test to assess the significance of frequency and continuous-variable differences between HA-VTE and non–HA-VTE groups. We investigated the association between each risk factor and HA-VTE using odds ratios (ORs) and 95% CIs based on univariate and multivariable regressions. We also investigated the association between HA-VTE events and 30-day readmission and 180-day mortality using Cox proportional hazard models. Missing data were noted and presented as such. Overall level of missing data for all risk factors among 1 098 171 admissions without HA-VTE was less than 2%, except for values of low hemoglobin (43 010 admissions with missing values [3.9%]), high platelet values (43 889 admissions [4.0%]), and high hemoglobin A_1c_ (ie, >10%; 533 262 admissions [48.6%]). For multivariate analyses, missing data were imputed to be negative or normal.

## Results

Admissions of 529 492 patients (268 797 women [50.8%]; median [IQR] age at time of first admission, 67.0 [54.0-79.0] years; 75 238 Asian [14.2%], 52 697 Black [10.0%], 79 398 Hispanic [15.0%], and 307 439 non-Hispanic White [58.1%]) were included in the study (eTable 3 in the Supplement). Among 1 112 014 medical admissions during the study period, 13 843 HA-VTE events (1.2%) were detected. Of these, 7946 were DVT events (57.4%), 4032 were PE events (29.1%), 1293 were DVT and PE events (9.3%), and 572 were of unknown VTE type (4.1%). Of all patients in the cohort, 10 410 individuals (2.0%) had at least 1 HA-VTE event during the study period (eTable 3 in the Supplement). Patients who experienced an HA-VTE were older, experienced more hospitalizations, were more likely to be Black or non-Hispanic White vs Asian or Hispanic, less likely to require an interpreter, and more likely to have received some pharmacological VTE prophylaxis in the first 48 hours of admission (eTable 3 in the Supplement).

**Figure 1.**
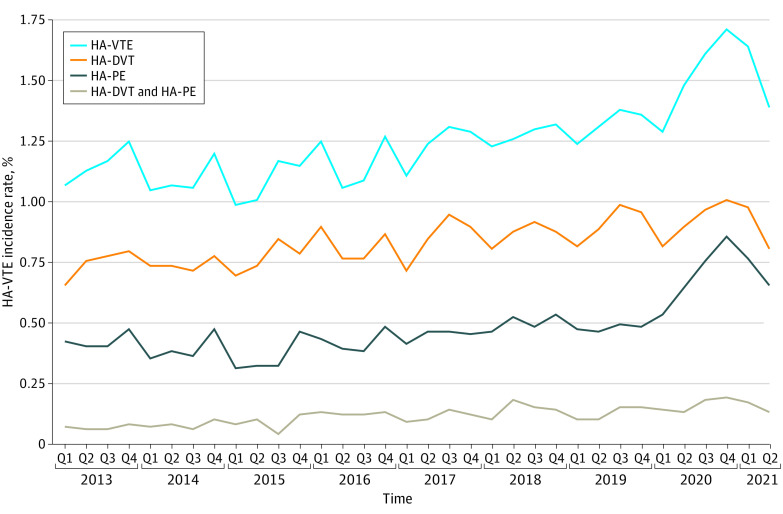
Incidence Rates Over Time The incidence of hospital-associated venous thromboembolism (HA-VTE) is given as the percentage of all included admissions. HA-DVT indicates hospital-associated deep vein thrombosis; HA-PE, hospital-associated pulmonary embolism; Q, quarter.

**Figure 2.**
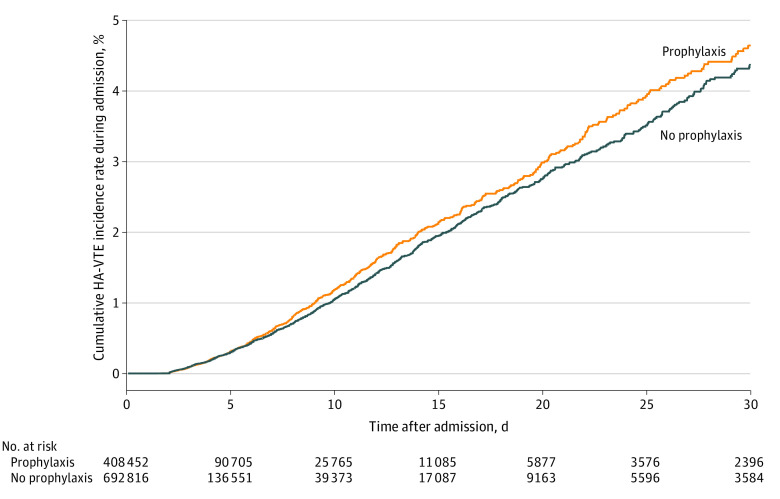
Time Course of Events During Admission The time course of hospital-associated venous thromboembolism (HA-VTE) events that happened during the index admission, with events censored at time of event or hospital discharge, is presented. Day 30 on the x-axis includes all hospitalizations lasting 30 or more days (5980 of 1 112 014 hospitalizations [0.5%]).

**Figure 3.**
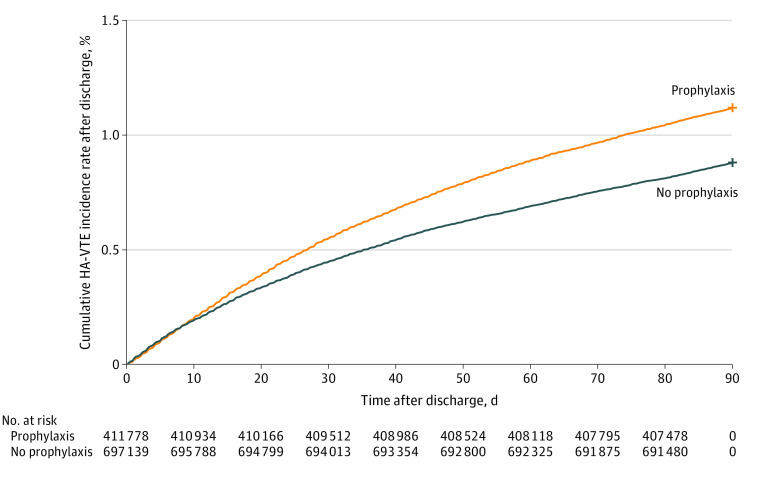
Time Course of Events After Discharge The time course of hospital-associated venous thromboembolism (HA-VTE) events that happened after discharge from the index admission, with events censored at 90 days after hospital discharge, is presented.

Over time, HA-VTE events increased from 307 of 29 095 admissions (1.1%) in the first quarter of 2013 to 551 of 33 729 admissions (1.6%) in the first quarter of 2021 (Figure 1). Most HA-VTE events occurred after discharge (10 746 events [77.6%]), while 3097 events (22.4%) occurred during the index admission. HA-VTE events were also more common in 413 062 admissions in which pharmacological VTE prophylaxis was given (5894 admissions [1.4%]) compared with 685 109 admissions in which prophylaxis was not given (7949 events [1.1%]), with higher odds of HA-VTE events in the subsets of patients with active cancer (adjusted OR [aOR], 1.28; 95% CI, 1.15-1.43), recent surgery (aOR, 1.37; 95% CI, 1.19-1.57), and reduced mobility (as defined in the eAppendix in the Supplement; aOR, 1.2; 95% CI, 1.02-1.42). Figure 2 shows cumulative incidence rates of HA-VTE during admission, with censoring at time of hospital discharge. HA-VTE cumulative incidence rates after hospital discharge are shown in Figure 3; eFigure 3 in the Supplement shows a histogram of the distribution of the number of days from discharge until detection of a HA-VTE event, and eFigure 4 in the Supplement shows the cumulative incidence of all HA-VTE events combined.

The Table displays baseline characteristics of patients and risk factors associated with HA-VTE at the hospitalization level, as well as associations between each risk factor and HA-VTE. In multivariable analysis, several factors were associated with increased odds of HA-VTE: active cancer (aOR, 1.96; 95% CI, 1.85-2.08), prior VTE (aOR, 1.71; 95% CI, 1.63-1.79), reduced mobility (aOR, 1.63; 95% CI, 1.50-1.77), presence of a PICC or an infusion port (aOR, 1.63; 95% CI 1.53-1.73), recent surgery or trauma (aOR, 1.5; 95% CI, 1.39-1.61), high platelet count (aOR, 1.34; 95% CI, 1.22-1.47), and low hemoglobin level (aOR, 1.29; 95% CI, 1.24-1.35). See additional statistically significant risk factors in the Table. Several other variables were associated with lower odds of HA-VTE: Asian race (vs non-Hispanic White race: aOR, 0.65; 95% CI, 0.61-0.69), current admission for stroke (aOR, 0.73; 95% CI, 0.65-0.81), Hispanic ethnicity (vs non-Hispanic White race: aOR, 0.81; 95% CI, 0.77-0.86), active tobacco smoking (aOR, 0.84; 95% CI, 0.78-0.89), current heart or respiratory failure (aOR, 0.85; 95% CI, 0.81-0.89), and recent use of a serotonergic antidepressant (aOR, 0.95; 95% CI, 0.92-0.99). Univariate analyses are also shown in the Table. In an analysis of factors potentially associated with odds of HA-VTE at the individual patient level, findings were similar (eTable 4 in the Supplement).

**Table.** Univariate and Multivariable Analyses of HA-VTE Risk Factors^a^

Variable	Patients, No./total (%)		Univariate regression		Multivariable regression	
	Admissions without HA-VTE (n = 1 098 171)	Admissions with HA-VTE (n = 13 843)	OR (95% CI)	*P* value	aOR (95% CI)	*P* value
Men	538 063 (49.0)	6873 (49.6)	1.03 (0.99-1.06)	.13	1.07 (1.03-1.1)	<.001
BMI						
>30	357 293 (33.1)	4873 (35.6)	1.12 (1.09-1.16)	<.001	1.12 (1.07-1.16)	<.001
Missing	20 192	170	NA	NA	NA	NA
Age at admission, median (IQR), y	71.0 (58.0, 81.0)	70.0 (59.0, 80.0)	1.00 (1.00-1.00)	.709	1.00 (1.00-1.00)	.009
Previous VTE	118 181 (10.8)	2610 (18.9)	1.93 (1.85-2.01)	<.001	1.71 (1.63-1.79)	<.001
Surgery or trauma in last 30 d	40 655 (3.7)	875 (6.3)	1.76 (1.64-1.88)	<.001	1.5 (1.39-1.61)	<.001
Active cancer	49 926 (4.5)	1517 (11.0)	2.58 (2.45-2.73)	<.001	1.96 (1.85-2.08)	<.001
Thrombophilia	10 211 (0.9)	205 (1.5)	1.6 (1.39-1.84)	<.001	1.22 (1.06-1.4)	.006
Current admission for infection	362 360 (33.0)	5135 (37.1)	1.2 (1.16-1.24)	<.001	1.07 (1.04-1.11)	<.001
Current admission for or with heart or respiratory failure	167 167 (15.2)	2039 (14.7)	0.96 (0.92-1.01)	.11	0.85 (0.81-0.89)	<.001
Current admission for stroke	39 978 (3.6)	327 (2.4)	0.64 (0.57-0.71)	<.001	0.73 (0.65-0.81)	<.001
Active smoker	101 930 (9.3)	1047 (7.6)	0.8 (0.75-0.85)	<.001	0.84 (0.78-0.89)	<.001
Diabetes						
Poorly controlled (Hg A_1c_ > 10%)	32 501 (3.0)	374 (2.7)	0.91 (0.82-1.01)	.075	0.91 (0.82-1.01)	.09
Missing	526 898	6364	NA	NA	NA	NA
Last Hg level at time of admission						
<10 g/dL	185 703 (16.9)	3345 (24.2)	1.57 (1.51-1.63)	<.001	1.29 (1.24-1.35)	<.001
Missing	42 541	469	NA	NA	NA	NA
Last platelet number at time of admission						
≥500 ×10^3^/uL	22 989 (2.1)	488 (3.5)	1.71 (1.56-1.87)	<.001	1.34 (1.22-1.47)	<.001
Missing	43 398	491	NA	NA	NA	NA
Major hemorrhage in last 30 d prior to admission	47 116 (4.3)	771 (5.6)	1.32 (1.22-1.41)	<.001	1.17 (1.09-1.26)	<.001
Any pharmacological VTE prophylaxis in first 48 h	407 168 (37.1)	5894 (42.6)	1.26 (1.22-1.30)	<.001	1.37 (1.32-1.41)	<.001
Race and ethnicity						
Asian	146 242 (13.3)	1230 (8.9)	0.63 (0.60-0.67)	<.001	0.65 (0.61-0.69)	<.001
Black	124 432 (11.3)	2105 (15.2)	1.40 (1.34-1.47)	<.001	1.21 (1.16-1.28)	<.001
Hispanic	153 596 (14.0)	1647 (11.9)	0.83 (0.79-0.87)	<.001	0.81 (0.77-0.86)	<.001
Non-Hispanic White	647 999 (59.0)	8594 (62.1)	1.14 (1.10-1.18)	<.001	1 [Reference]	NA
Other or unknown	25 902 (2.4)	267 (1.9)	0.81 (0.72-0.92)	<.001	0.81 (0.72-0.92)	.001
Requires interpreter	45 458 (4.1)	423 (3.1)	0.73 (0.66-0.80)	<.001	0.95 (0.85-1.05)	.32
COPS ≥ 50	445 802 (40.6)	6775 (48.9)	1.40 (1.36-1.45)	<.001	1.05 (1.01-1.09)	.01
LAPS ≥ 100	206 152 (18.8)	3241 (23.4)	1.32 (1.27-1.38)	<.001	1.22 (1.17-1.27)	<.001
Proteinuria or microalbuminuria	52 998 (4.8)	645 (4.7)	0.96 (0.89-1.04)	.363	0.94 (0.86-1.02)	.11
PICC line or infusion port at admission	45 379 (4.1)	1323 (9.6)	2.45 (2.31-2.60)	<.001	1.63 (1.53-1.73)	<.001
Antidepressant	266 742 (24.3)	3536 (25.5)	1.07 (1.03-1.11)	<.001	0.95 (0.92-0.99)	.02
Hormonal treatment	31 020 (2.8)	462 (3.3)	1.19 (1.08-1.30)	<.001	1.05 (0.95-1.15)	.36
Reduced mobility	29 488 (2.7)	649 (4.7)	1.78 (1.64-1.93)	<.001	1.63 (1.50-1.77)	<.001

Abbreviations: aOR, adjusted odds ratio; BMI, body mass index (calculated as weight in kilograms divided by height in meters squared); COPS, Comorbidity Point Score; HA-VTE, hospital-associated venous thromboembolism; Hg, hemoglobin; LAPS, Laboratory Acute Physiology Score; NA, not applicable; OR, odds ratio; PICC, peripherally inserted central; VTE, venous thromboembolism.

SI conversion factors: To convert Hg level to grams per liter, multiply by 10.0; Hg A_1c_ to proportion of total hemoglobin, multiply by 0.01; platelet count to ×10^9^ per liter, multiply by 1.0.

^a^ Analyses were at the admission level.

**Figure 4.**
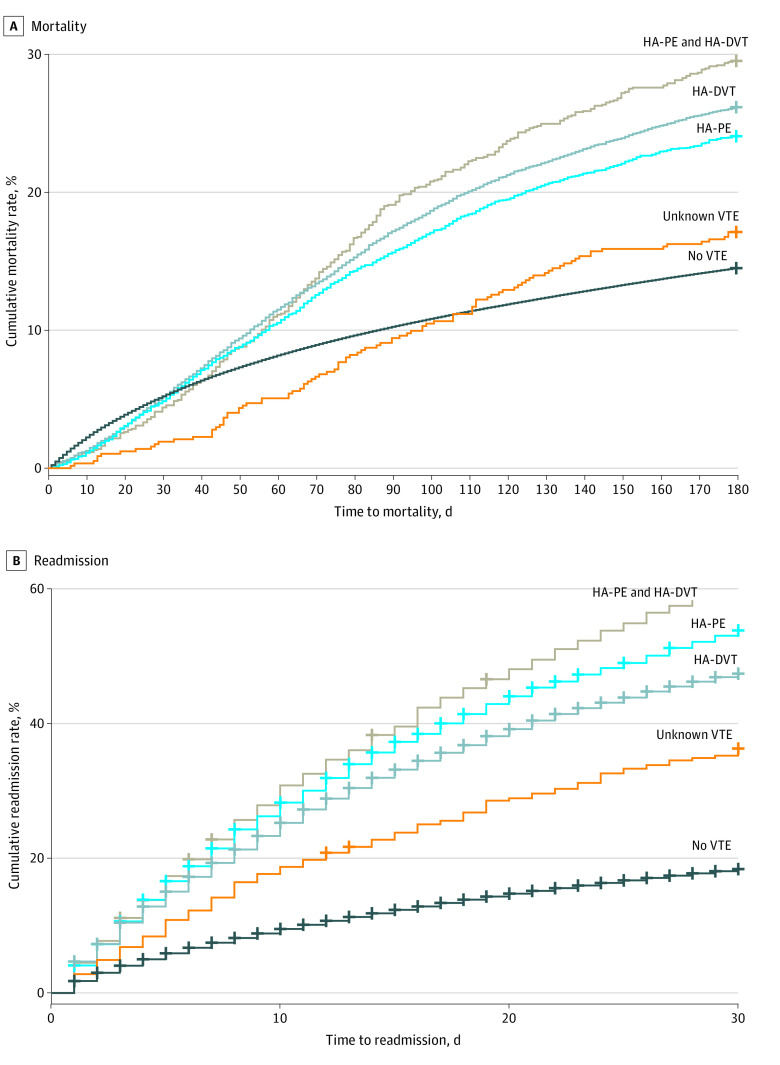
Mortality and Readmission Rates Kaplan-Meier survival curves among patients with and without hospital-associated venous thromboembolism (HA-VTE) events are presented for A, mortality (day 0 is day of admission) and B, readmissions (day 0 is day of discharge). HA-DVT indicates hospital-associated deep vein thrombosis; HA-PE, hospital-associated pulmonary embolism.

HA-VTE events were associated with increased risk of readmission (hazard ratio [HR], 3.33; 95% CI, 3.25-3.41) and mortality (HR, 1.63; 95% CI, 1.57-1.70). Comparing clinical outcomes of patients who had various subclassifications of HA-VTE with those among patients without HA-VTE, all HA-VTE events were associated with increased hazards for 180-day mortality (HA-DVT and PE: HR, 2.14; 95% CI, 1.94-2.37; HA-DVT: HR, 1.88; 95% CI, 1.8-1.97; HA-PE: HR, 1.72; 95% CI, 1.61-1.83), except for unknown HA-VTE type (HR, 1.17; 95% CI, 0.96-1.42). Findings were similar for readmission within 30 days from discharge (HA-DVT and PE: HR, 4.29; 95% CI, 3.99-4.60; HA-PE: HR, 3.67; 95% CI, 3.51-3.83; HA-DVT: HR, 3.11; 95% CI, 3.01-3.21; HA-VTE of unknown subtype: HR, 2.19; 95% CI, 1.91-2.50) (Figure 4; eFigure 5 and eFigure 6 in the Supplement).

## Discussion

In this cohort study, we examined a large and contemporary cohort of patients with medical hospitalizations in a highly integrated multicenter health care system. Our findings were broadly consistent with those of previous reports. For example, our 1.2% HA-VTE incidence rate was similar to a 1.29% rate in a single-center study from Vermont between 2010 and 2016^40^ and a 0.97% rate between 2010 and 2013 in a 2018 study from Australia^41^; however, these studies relied mostly or solely on *ICD-9* and *ICD-10* codes, a methodology that has been repeatedly shown to have high rates of false-positive and false-negative events.^15,16,17,18^ We also observed an increase in HA-VTE events in recent years, which is concordant with results from a prior study^42^ showing that VTE events (without distinguishing HA-VTE from VTE) have increased since 2001, as well as an apparent temporary increase since the COVID-19 pandemic began.^43,44^ Importantly, this apparent increase in incidence may partially be explained by improved diagnostic technologies and clinical workflows over the years. Similar to previous reports,^7,42^ our findings showed markedly worse clinical outcomes associated with HA-VTE, including a significant increase in mortality and readmission rates. However, it may be that other variables associated with HA-VTE (eg, length of hospitalization) underlie the association between HA-VTE and these clinical outcomes. Additionally, a meta-analysis of 21 randomized clinical trials^13^ found that while pharmacological prophylaxis was associated with reduced HA-VTE, this was not associated with lower mortality, somewhat limiting the clinical implications of these findings. Finally, similar to prior studies,^16,40,41,45^ we found that most HA-VTE events happened after discharge and that the increased risk of VTE after admission decreased over time but did not plateau by 90 days after discharge.

Additionally, consistent with previous reports, we found that cancer,^24^ prior VTE,^24^ immobilization,^24^ an indwelling PICC line or infusion port,^32^ surgery or trauma,^24^ thrombocytosis,^31^ anemia,^29,30^ thrombophilia,^24^ Black race,^37^ recent major hemorrhage,^36^ obesity,^24^ male sex,^38^ and acute infection were associated with HA-VTE risk.^24^ In addition, we found in this study that the automatically captured illness severity indices COPS and LAPS^35^ were associated with HA-VTE. Unexpectedly, we found that a few risk factors previously reported to be associated with HA-VTE^24,25,26,33^ were associated with decreased risk; these included acute stroke, tobacco smoking, acute respiratory or heart failure, and serotonergic antidepressant use. These discrepancies were likely associated with residual confounding.

We additionally found that thromboprophylaxis given in the first 48 hours of admission was associated with an increased risk of HA-VTE in the overall study population and specific patient subsets (those with active cancer, recent surgery, or reduced mobility). This is likely associated with the more common use of prophylaxis among patients with greater baseline risk of HA-VTE and, as others have suggested,^46^ the likelihood that clinicians who give more prophylaxis will order diagnostic studies for HA-VTE. Notably, this increase in HA-VTE risk among patients receiving prophylaxis was lower during the first week of admission compared with events after discharge. This may suggest, as others have noted,^22^ that prophylaxis given in the hospital is mostly associated with a decrease in HA-VTE events that happen during the admission.

Our study has several strengths. First, to our knowledge, this is the largest contemporary cohort of medical patients with HA-VTE, including the largest number of explored risk factors associated with HA-VTE within a single study. Second, our HA-VTE algorithm was designed to account for many methodological challenges faced by other studies on HA-VTE.^7,15,16,17,18,19,20^ Our logic requiring either a definitive PE diagnosis on CT or a combination of diagnosis and treatment indications, as well as the exclusion of admissions in which there was an HA-VTE event or administration of therapeutic anticoagulation in the first 48 hours, appears to have been associated with more accurate detection of true HA-VTE than reliance on diagnosis codes alone.^16,22^ The inclusion of HA-VTE up to 90 days after discharge may have been especially important given findings by us and others^15,16^ that most HA-VTE events happened after discharge. Additionally, unlike other studies,^20,22,47^ to better simulate a common clinical scenario of clinician risk assessment at time of admission and reduce various forms of biases, our algorithm was permitted to capture only data associated with risk factors as they were recorded at the exact time of admission. Furthermore, because KPNC is an integrated health care delivery system, its EHRs contain almost all member health data, reducing the chances for inaccurate identification of risk factors and outcomes. Taken together, these strengths may be associated with not only improved accuracy in our cohort, but also improved validity of findings on risk factors, incidence rates, time course, and clinical outcomes associated with HA-VTE. Additionally, the community hospital setting and our diverse patient population^21^ may be associated with improved external validity for our findings, and the distinction between subtypes of HA-VTE may be associated with improved clinical relevance.

Our use of detailed EHR data for risk characterization may additionally serve several purposes in future research. These may include assessments related to HA-VTE quality initiatives, as well as the development and validation of real-time risk-assessment models and clinical decision–support tools to help quantify and reduce HA-VTE risk at time of admission. The importance of such tools has been increasingly recognized, with reports in 2021^3,22^ and 2016^39^ of lower than expected performance of established manual risk-assessment models.

### Limitations

Our study has some important limitations. First, while we accounted for minor data structuring changes during our study period, there may have been changes in sensitivity of imaging tests, relevant workflows, and documentation practices over time (eg, accuracy of *ICD-9* and *ICD-10* codes and recent introduction of tags in our CT radiology reports), potentially creating a bias in estimating HA-VTE trends over time. Second, we excluded patients who received therapeutic doses of anticoagulation in the first 48 hours of admission, which precluded detection of HA-VTE events that happened despite anticoagulation. Third, we included only hospitalizations within KPNC, which could have resulted in underestimation or overestimation of HA-VTE rates. Fourth, while our HA-VTE detection algorithm may inspire similar work in other health care systems, expected differences in data structuring and ownership would likely limit replicability.

## Conclusions

HA-VTE in medical patients remains one of the most burdensome hospital-associated complications, but, fortunately, it is highly preventable.^7^ This cohort study’s findings offer a contemporary and reliable description of patient characteristics and admissions associated with this outcome. Further research is needed to develop more accurate risk-assessment tools, which would preferably be automatic and available in real time given that available models can be time consuming^48^ and inadequately predictive.^3,22,39^ Accurate automatic and dynamic capture of HA-VTE may be an important first step in this direction.

## Notes

Supplement. **eFigure 1.** Diagram of Venous Thromboembolism Events Considered Hospital Acquired According to Time of Diagnosis **eFigure 2.** Diagram of Criteria and Logic for Determination of Hospital-Acquired Venous Thromboembolism Events **eFigure 3.** Histogram of No. of Days Between Discharge and Detection of Hospital-Acquired Venous Thromboembolism Event **eFigure 4.** Time Course of Hospital-Acquired Venous Thromboembolism Events Occurring During Admission or After Discharge **eFigure 5.** Mortality Rates With No. at Risk **eFigure 6.** Readmission Rates With No. at Risk **eTable 1.** Prevalence of Specific Hospital-Acquired Venous Thromboembolism Definition Criteria Items **eTable 2.** Distributions of Total Numbers of Positive Criteria for Hospital-Acquired Venous Thromboembolism in Admissions With and Without Associated Venous Thromboembolism **eTable 3.** Baseline Demographics of Patients With and Without Venous Thromboembolism **eTable 4.** Multivariable Analysis of Risk Factors Associated With Hospital-Acquired Venous Thromboembolism **eAppendix.** Definitions of Variables
