# Supplementary material for: Trends and Risk Factors for Venous Thromboembolism Among Hospitalized Medical Patients
Source: JAMA Netw Open. 2022 Nov 21;5(11):e2240373. doi: 10.1001/jamanetworkopen.2022.40373 (PMC9679881; doi:10.1001/jamanetworkopen.2022.40373)
Supplement: Supplement. — eFigure 1. Diagram of Venous Thromboembolism Events Considered Hospital Acquired According to Time of Diagnosis eFigure 2. Diagram of Criteria and Logic for Determination of Hospital-Acquired Venous Thromboembolism Events eFigure 3. Histogram of No. of Days Between Discharge and Detection of Hospital-Acquired Venous Thromboembolism Event eFigure 4. Time Course of Hospital-Acquired Venous Thromboembolism Events Occurring During Admission or After Discharge eFigure 5. Mortality Rates With No. at Risk eFigure 6. Readmission Rates With No. at Risk eTable 1. Prevalence of Specific Hospital-Acquired Venous Thromboembolism Definition Criteria Items eTable 2. Distributions of Total Numbers of Positive Criteria for Hospital-Acquired Venous Thromboembolism in Admissions With and Without Associated Venous Thromboembolism eTable 3. Baseline Demographics of Patients With and Without Venous Thromboembolism eTable 4. Multivariable Analysis of Risk Factors Associated With Hospital-Acquired Venous Thromboembolism eAppendix. Definitions of Variables [file jamanetwopen-e2240373-s001.pdf]

Supplemental Online Content

Neeman E, Liu V, Mishra P, et al. Trends and risk factors for venous thromboembolism among hospitalized medical patients. *JAMA Netw Open*. 2022;5(11):e2240373.
doi:10.1001/jamanetworkopen.2022.40373

eFigure 1. Diagram of Venous Thromboembolism Events Considered Hospital Acquired According to Time of Diagnosis

eFigure 2. Diagram of Criteria and Logic for Determination of Hospital-Acquired Venous Thromboembolism Events

eFigure 3. Histogram of No. of Days Between Discharge and Detection of Hospital-Acquired Venous Thromboembolism Event

eFigure 4. Time Course of Hospital-Acquired Venous Thromboembolism Events Occurring During Admission or After Discharge

eFigure 5. Mortality Rates With No. at Risk

eFigure 6. Readmission Rates With No. at Risk

eTable 1. Prevalence of Specific Hospital-Acquired Venous Thromboembolism Definition Criteria Items

eTable 2. Distributions of Total Numbers of Positive Criteria for Hospital-Acquired Venous Thromboembolism in Admissions With and Without Associated Venous Thromboembolism

eTable 3. Baseline Demographics of Patients With and Without Venous Thromboembolism

eTable 4. Multivariable Analysis of Risk Factors Associated With Hospital-Acquired Venous Thromboembolism

eAppendix. Definitions of Variables

This supplemental material has been provided by the authors to give readers additional information about their work.

© 2022 Neeman E et al. *JAMA Network Open.*

eFigure 1. Diagram of Venous Thromboembolism Events Considered Hospital Acquired According to Time of Diagnosis

Pre-existing VTE				HA-VTE			Unrelated VTE
Prior to admission		Day 0 of admission		48 hours post admission	Rest of admission	Day of discharge	90 days post discharge

© 2022 Neeman E et al. *JAMA Network Open.*

eFigure 2. Diagram of Criteria and Logic for Determination of Hospital-Acquired Venous Thromboembolism Events

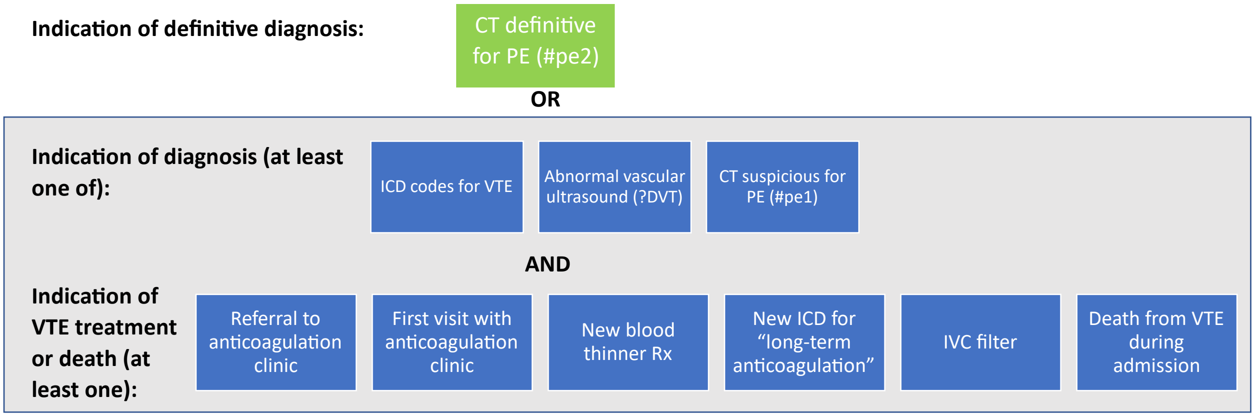

© 2022 Neeman E et al. *JAMA Network Open.*

eFigure 3. Histogram of No. of Days Between Discharge and Detection of Hospital-Acquired Venous Thromboembolism Event

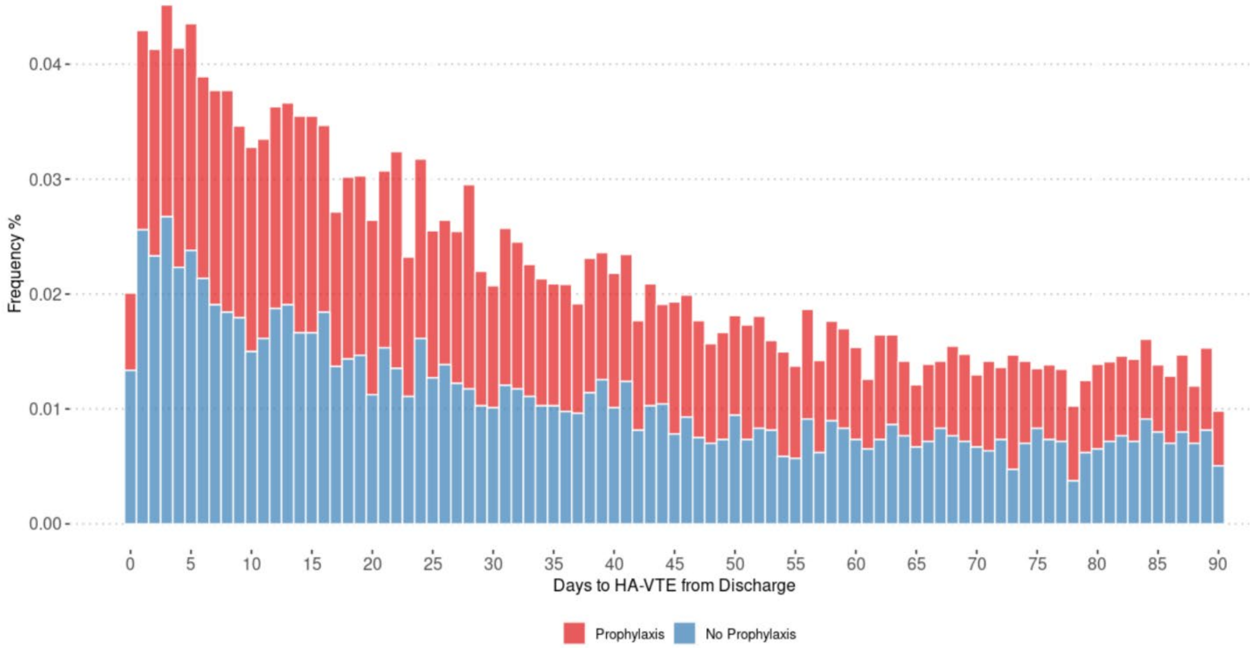

eFigure 3 legend: histogram depicting the distribution of the number of days from discharge until detection of a HA-VTE event

© 2022 Neeman E et al. *JAMA Network Open.*

eFigure 4. Time Course of Hospital-Acquired Venous Thromboembolism Events Occurring During Admission or After Discharge

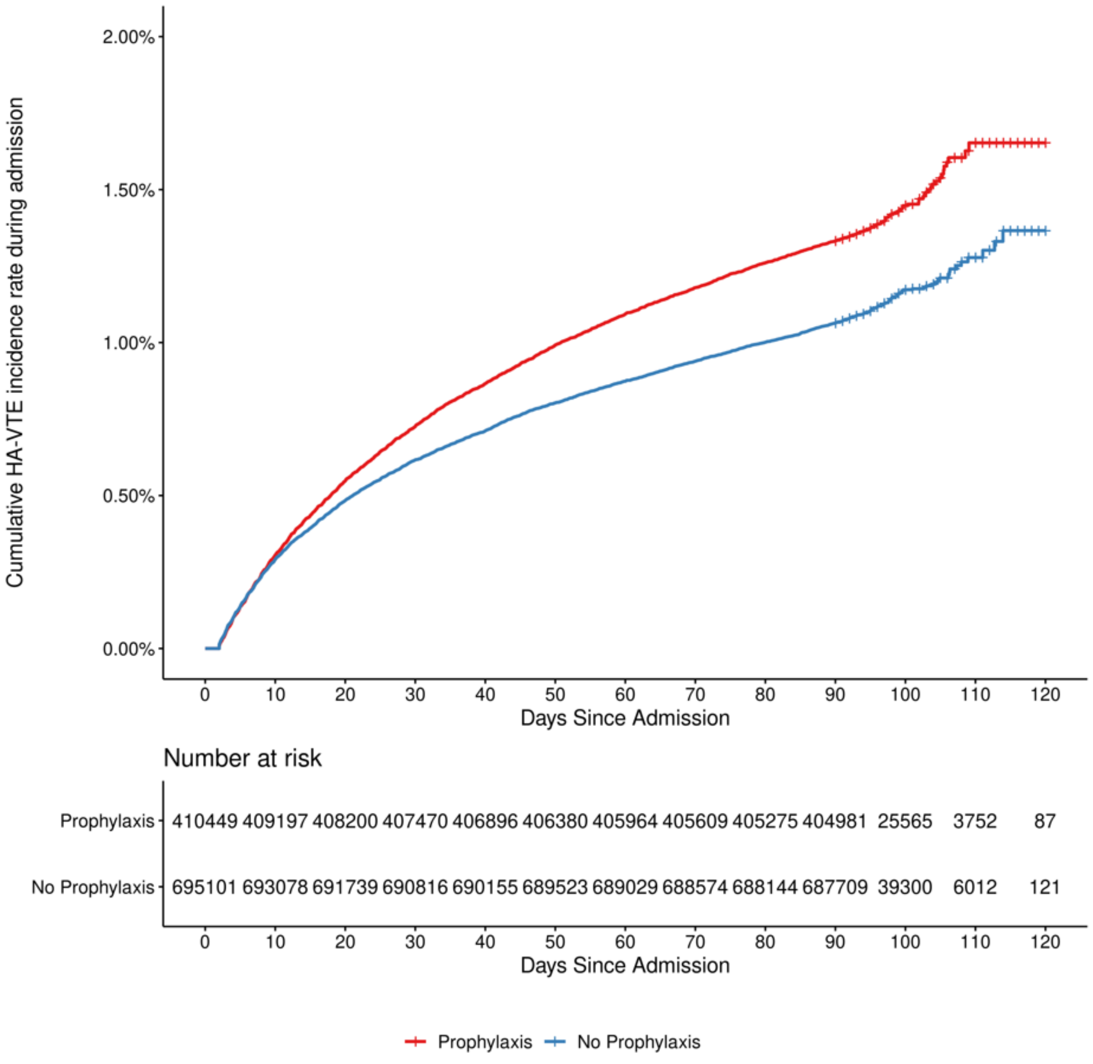

eFigure 4 legend: time course of HA-VTE events that happened during the index admission or after discharge, with cases censored at 90 days after discharge

© 2022 Neeman E et al. *JAMA Network Open.*

eFigure 5. Mortality Rates With No. at Risk

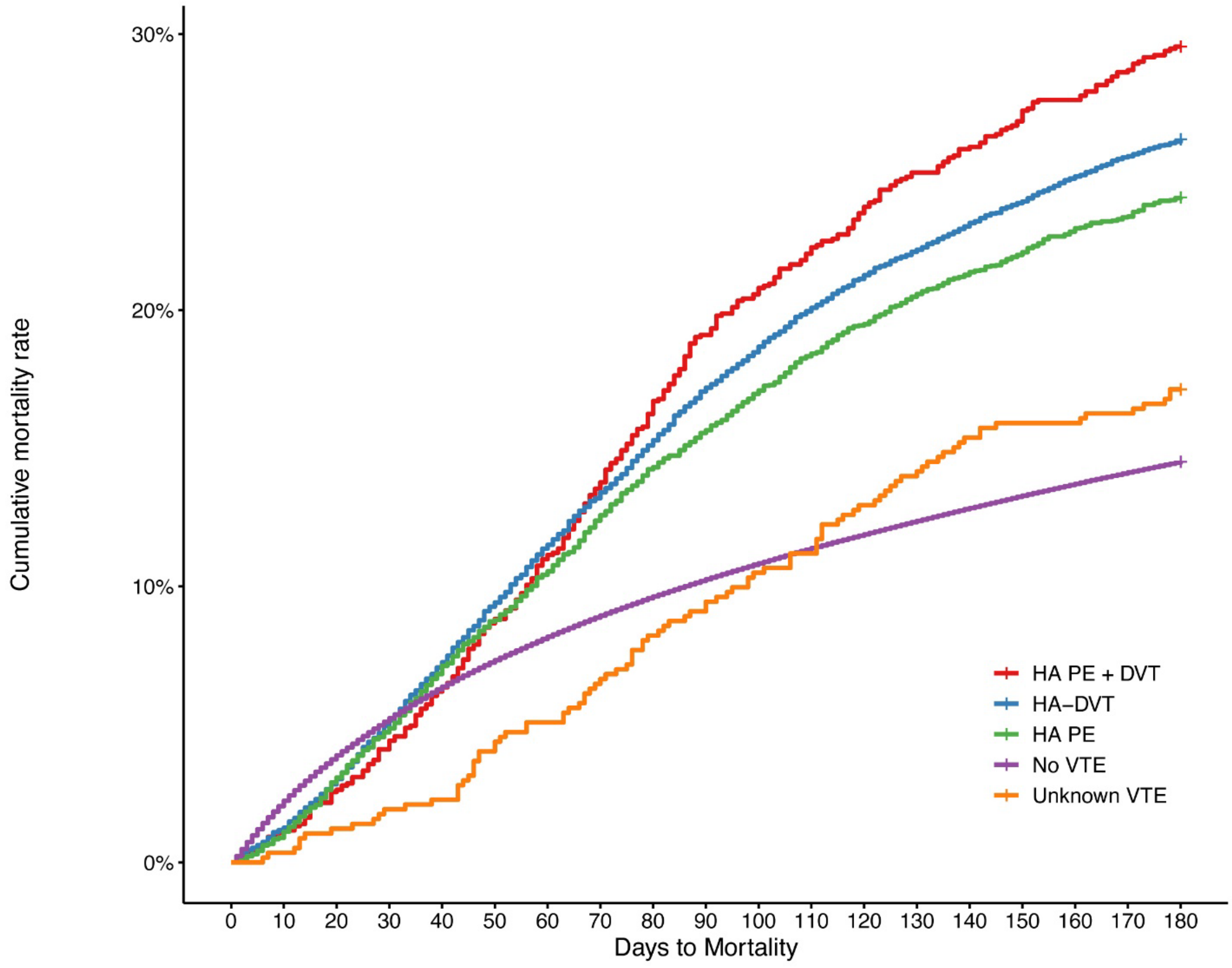

Number at risk

Days to Mortality	0	10	20	30	40	50	60	70	80	90	100	110	120	130	140	150	160	170	180
HA PE + DVT	1293	1279	1260	1240	1213	1181	1151	1118	1083	1046	1027	1008	989	970	959	946	936	923	911
HA–DVT	7946	7853	7721	7553	7385	7210	7043	6898	6746	6591	6478	6360	6267	6189	6116	6049	5978	5917	5872
HA PE	4032	3996	3915	3842	3757	3680	3612	3533	3458	3405	3348	3293	3248	3206	3174	3145	3111	3090	3062
No VTE	1098171	1075792	1057182	1042238	1029439	1018738	1009322	1000815	993117	986257	979809	973783	968266	962862	957713	952780	948062	943518	939209
Unknown VTE	572	570	565	561	559	549	543	535	525	520	512	508	498	492	484	481	481	479	474

© 2022 Neeman E et al. *JAMA Network Open.*

eFigure 6. Readmission Rates With No. at Risk

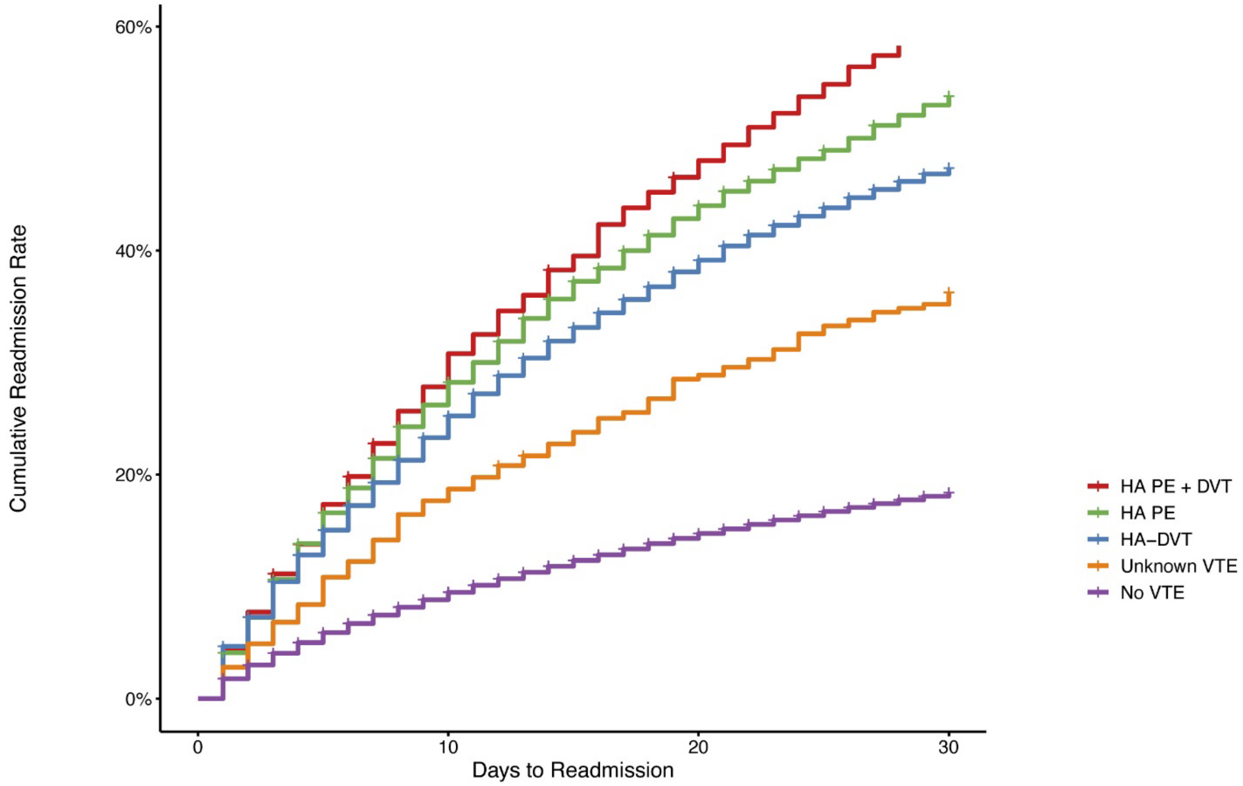

Number at risk

	0	10	20	30
HA PE + DVT	1293	927	683	517
HA PE	4032	2954	2272	1861
HA–DVT	7946	6038	4842	4127
Unknown VTE	572	471	407	369
No VTE	1098171	983003	913129	865960

Days to Readmission

© 2022 Neeman E et al. *JAMA Network Open.*

eTable 1. Prevalence of Specific Hospital-Acquired Venous Thromboembolism Definition Criteria Items

HA-VTE criterion	Admissions with this criterion: n (%)	Admissions with this criterion within admissions resulting in HA-VTE: n (%)	Admissions with this criterion within admissions not resulting in HA-VTE: n (%)
VTE ICD codes	19,156 (1.72%)	13,000 (93.91%)	6,156 (0.56%)
Abnormal vascular US	8,443 (0.76%)	5,687 (41.08%)	2,756 (0.25%)
CT with possible PE (#PE1)	255 (0.02%)	66 (0.48%)	189 (0.02%)
CT with definite PE (#PE2)	261 (0.02%)	261 (1.89%)	(0%)
Referral to anticoagulation clinic	9,494 (0.85%)	7,417 (53.58%)	2,077 (0.19%)
First visit with anticoagulation clinic	8,530 (0.77%)	7,378 (53.30%)	1,152 (0.1%)
New medication for VTE	31,384 (2.82%)	10,347 (74.75%)	21,037 (1.92%)
ICD for long term anticoagulation	16,239 (1.46%)	3,879 (28.02%)	12,360 (1.13%)

© 2022 Neeman E et al. *JAMA Network Open.*

IVC filter	1,685 (0.15%)	1,346 (9.72%)	339 (0.03%)
In hospital death after receiving anticoagulation	746 (0.07%)	255 (1.84%)	491 (0.04%)

© 2022 Neeman E et al. *JAMA Network Open.*

eTable 2. Distributions of Total Numbers of Positive Criteria for Hospital-Acquired Venous Thromboembolism in Admissions With and Without Associated Venous Thromboembolism

**Number of positive HA-VTE criteria**	**Admissions resulting in HA-VTE: n (%)**	**Admissions not resulting (without) HA-VTE: n (%)**
0	0 (0%)	1,059,224 (96.45%)
1	10 (0.07%)	30,457 (2.77%)
2	4,260 (30.77%)	7,751 (0.71%)
3	2,436 (17.6%)	591 (0.05%)
4	2,983 (21.55%)	145 (0.01%)
5	3,125 (22.57%)	3 (0%)
6	964 (6.96%)	0 (0%)
7	63 (0.46%)	0 (0%)
8	2 (0.01%)	0 (0%)
9-10	0 (0%)	0 (0%)
Total	13,843 (100.00%)	1,098,171 (100.00%)

© 2022 Neeman E et al. *JAMA Network Open.*

eTable 3. Baseline Demographics of Patients With and Without Venous Thromboembolism

Analysis is at the patient level.

Variable	Entire cohort (N = 529,492)/ n (%)	Patients with no HA-VTE (N =519,082)/ n (%)	Patients with HA-VTE (N = 10,410)/ n (%)	p value
Legal sex at time of admission				0.6
Male	260,640 (49.2%)	255,488 (49.2%)	5,152 (49.5%)	
Female	268,797 (50.8%)	263,539 (50.8%)	5,258 (50.5%)	
Missing	55	55	0	
Median age at time of first admission in study period - Median (IQR)	67.0 (54.0, 79.0)	67.0 (54.0, 79.0)	70.0 (59.0, 80.0)	<0.001
Number of admissions in study period - Median (IQR)	1.0 (1.0, 2.0)	1.0 (1.0, 2.0)	3.0 (2.0, 6.0)	<0.001
Race/Ethnicity				
Asian	75,238 (14.2%)	74,328 (14.3%)	910 (8.7%)	<0.001
Black	52,697 (10.0%)	51,186 (9.9%)	1,511 (14.5%)	

© 2022 Neeman E et al. *JAMA Network Open.*

Hispanic	79,398 (15.0%)	78,165 (15.1%)	1,233 (11.8%)	
Other/Unknown	14,720 (2.8%)	14,515 (2.8%)	205 (2.0%)	
White	307,439 (58.1%)	300,888 (58.0%)	6,551 (62.9%)	
Requires interpreter (%yes)	24,766 (4.7%)	24,443 (4.7%)	323 (3.1%)	<0.001
Received prophylaxis in first 48h	200,323 (37.8%)	195,871 (37.7%)	4,452 (42.8%)	<0.001

© 2022 Neeman E et al. *JAMA Network Open.*

eTable 4. Multivariable Analysis of Risk Factors Associated With Hospital-Acquired Venous Thromboembolism

Analysis is at the admission level.

Variable	Odds Ratio	95% CI	p-value
Male	1.07	1.01,1.13	0.02
BMI >30	1.13	1.06,1.2	<0.001
Age at time of admission (Median, IQR)	1.00	1,1	0.08
Previous VTE	1.88	1.73,2.05	<0.001
Surgery/trauma last 30d	1.48	1.31,1.67	<0.001
Active cancer	1.87	1.68,2.09	<0.001
Thrombophilia	1.47	1.15,1.88	<0.001
Current admission for infection	1.07	1.01,1.14	0.02
Current admission for/with heart or respiratory failure	0.92	0.84,1	0.05
Current admission for stroke	0.67	0.57,0.8	<0.001
Active smoker	0.81	0.73,0.9	<0.001
Poorly controlled DM (A1c>10%)	0.98	0.81,1.18	0.82
Last Hg at time of admission <10 g/dL	1.40	1.29,1.51	<0.001
Last platelet number at time of admission >=500,000/uL	1.59	1.37,1.84	<0.001
Major hemorrhage in last 30d prior to admission	1.13	0.99,1.31	0.08
Any pharmacological VTE prophylaxis in first 48h	1.30	1.22,1.38	<0.001

© 2022 Neeman E et al. *JAMA Network Open.*

Asian	0.63	0.57,0.7	<0.001
Black	1.20	1.1,1.31	<0.001
Hispanic	0.75	0.68,0.82	<0.001
Other/Unknown	0.72	0.58,0.88	<0.001
Requires interpreter	1.01	0.86,1.18	0.94
COPS ≥50	1.12	1.05,1.2	0.001
LAPS ≥100	1.32	1.23,1.42	<0.001
Proteinuria/micro-albuminuria	0.99	0.86,1.14	0.86
PICC line or infusion port at admission	1.73	1.54,1.94	<0.001
Antidepressant	0.93	0.87,1	0.05
Hormonal Treatment	1.00	0.85,1.17	0.96
Reduced Mobility	1.85	1.63,2.11	<0.001

© 2022 Neeman E et al. *JAMA Network Open.*

eAppendix. Definitions of Variables

In all cases where International Classification of Diseases (ICD) codes were used, both ICD 9 and 10 versions were included.

HA-VTE Outcome

Theoretical definition: any new diagnosis of VTE starting at least 48h after initiation of index admission and up to 90 days after discharge.

Operational definition: Logic: “true” if any of the following criteria combinations are met:

- (1 AND 2); or
- 4; or
- (1 OR 2 OR 3) AND (5 OR 6 OR 7 OR 8 OR 9 OR 10)

HA-VTE Criteria definitions:

1. Theoretical definition: ICD codes describing HA-VTE events

   Operational definition: “true” if one of these ICD9 or ICD10 codes were first entered as an encounter diagnosis or in problem list at least 48h after initiation of index admission and up to 90 days after discharge.

   - ICD 9: 415.1, 415.11, 415.19, 451.1, 451.11, 453.2, 453.2, 453.3, 453.8, 453.9, 453.4, 453.41, 453.42, 453.40, 453.41, 453.82, 453.83, 453.84, 453.85, 453.89
   - ICD 10: I26.0; I26.9; I80.1; I80.2; I80.3; I80.8; I80.9;I82.2; I82.4; I82.6; I82.8; I82.9; I82.A1; I82.B1; I82.C1; O22.3; O87.1; O88.2;

© 2022 Neeman E et al. *JAMA Network Open.*

If it is not possible to distinguish when during the admission the ICD code was entered, then include any of these ICD codes if they were started (first entered) DURING the admission and up to 90 days after discharge

2. Theoretical definition: vascular ultrasound showing HA-VTE
   Operational definition: "true" if "Abnormal" result or flag ("!") present for vascular ultrasound with test date/time (*defined in this case as the date and time labeled "exam end")* is at least 48h after initiation of index admission and up to 90 days after discharge

3. Theoretical definition: CT pulmonary angiogram showing possible HA-VTE
   Operational definition: "true" if a CT pulmonary report with the text string "#pe1" in it, with test date/time (*time labeled "exam end")* at least 48h after initiation of index admission and up to 90 days after discharge.
   note: #pe1 is a tag added to CT reports by radiologists to denote a suspicion for PE. It was introduced to KPNC in 06/2020 and is not universally adopted.

4. Theoretical definition: CT pulmonary angiogram showing definite HA-VTE
   Operational definition: "true" if a CT pulmonary angiogram report with the text string "#pe2" in it, with test date/time (*time labeled "exam end")* at least 48h after initiation of index admission and up to 90 days after discharge
   note: #pe2 is a tag added to CT reports by radiologists to denote a definitive diagnosis of PE. It was introduced to KPNC in 06/2020 and is not universally

© 2022 Neeman E et al. *JAMA Network Open.*

adopted.

5. Theoretical definition: a referral to anticoagulation clinic for HA-VTE

   Operational definition: “true” if an eConsult (i.e., internal referral via proprietary referral system at KPNC) to anticoagulation clinic with any of the following Problem/Reason Codes (PRCs) was placed at least 48h after initiation of index admission and up to 90 days after discharge

   - “DVT/PE-VTE (warfarin)” *this PRC existed and has stayed the same since 4/16/2003*
   - “DVT/PE - VTE (DOAC)” *this PRC existed and has stayed the same since 4/9/2015*

6. Theoretical definition: a visit with anticoagulation clinic for HA-VTE

   Operational definition: “true” if:

   - No prior anticoagulation clinic encounter within 180 days prior to index admission AND
   - Has an anticoagulation clinic encounter within 90 days or less after date of discharge from index admission (see definition and example below) AND
   - The first anticoagulation clinic Progress Note after discharge from index admission includes any of the following verbiage anywhere in the note: “VTE” OR “PE” OR “DVT” or “deep vein thrombosis” or “pulmonary embolism” (not case sensitive).

© 2022 Neeman E et al. *JAMA Network Open.*

7. Theoretical definition: a new medication given to treat HA-VTE

   Operational definition: “true” if: first order for one of the drugs below is signed at least 48 hours after initiation of index admission and within 90 days from date of discharge AND none of these drugs below were filled for at least 1 year prior to index admission (internal pharmacy codes were used to identify all relevant formulations of the drugs below):

   - Warfarin any dose/regimen
   - Apixaban any dose/regimen
   - Rivaroxaban any dose/regimen
   - Dabigatran any dose/regimen
   - Enoxaparin if given twice or three times a day at any dose>30mg, or if given once daily with dose being >0.8mg/kg where kg is based on the most recent weight prior to the enoxaparin order

8. Theoretical definition: an ICD code denoting that the patient is on a long-term anticoagulant

   Operational definition: ICD10 code Z79.01, ICD 9 code V58.61: “true” if one of these codes first entered at least 48h after initiation of index admission and up to 90 days after discharge. Exclude if the same code was previously entered or existed in the problem list (unresolved problem) in the 6 months prior to index admission.

   If it is not possible to distinguish when during the admission the ICD code was entered, then include any of these ICD codes if they were started (first entered)

© 2022 Neeman E et al. *JAMA Network Open.*

DURING the admission and up to 90 days after discharge

9. Theoretical definition: an IVC filter was inserted as a substitute for anticoagulation as treatment for HA-VTE
   Operational definition: "true" if CPT code 37191 OR ICD10 code Z95.828 OR ICD-9-CM procedure code 38.7 first entered at least 48h after initiation of index admission and up to 90 days after discharge
   If it is not possible to distinguish when during the admission the ICD code was entered, then include any of these ICD codes if they were started (first entered) DURING the admission and up to 90 days after discharge

10. Theoretical definition: a patient who died during the index admission after receiving therapeutic anticoagulation
   Operational definition: "true" if: the patient died DURING the index admission AND an order set relating to therapeutic anticoagulation was used during this index admission

Definitions of HA-VTE subtypes

For each admission that has a positive HA-VTE outcome, determine whether they had HA DVT, HA PE or both

1. HA DVT
   a. Theoretical definition: any new diagnosis of HA DVT starting at least 48h after initiation of index admission and up to 90 days after discharge
   b. Operational definition: in patients with HA-VTE as defined above:

i. If outcome criterion #2 (abnormal US) is positive: code as true (1); otherwise:

ii. If outcome criterion #1 (ICD codes) included any of the following ICD codes for DVT, code as true;

1. ICD 9: 451.1, 451.11, 453.2, 453.3, 453.8, 453.9, 453.4, 453.41, 453.42, 453.40, 453.82, 453.83, 453.84, 453.85, 453.89
2. ICD 10: I80.1; I80.2; I80.3; I80.8; I80.9;I82.2; I82.4; I82.6; I82.8; I82.9; I82.A1; I82.B1; I82.C1; O22.3; O87.1; O88.2;

iii. If (i) and (ii) above are not true, code as false (0)

2. HA PE

a. Theoretical definition: any new diagnosis of HA PE starting at least 48h after initiation of index admission and up to 90 days after discharge

b. Operational definition: in patients with HA-VTE:

i. If outcome criterion #3 (#pe1) OR outcome criterion #4 (#PE2) is positive: code as true (1); else,

ii. If outcome criterion #1 (ICD codes) included any of the following ICD codes for DVT, code as true;

1. ICD9: 415.1, 415.11, 415.19
2. ICD10: I26.0; I26.9;

iii. If (i) and (ii) above are not true, code as false (0)

© 2022 Neeman E et al. *JAMA Network Open.*

Determining timing of HA-VTE event

1. Time to first VTE from admission
    a. Theoretical definition: number of days since admission time to time of first HA-VTE event (if it occurred)
    b. Operational definitions:
        i. Time of HA-VTE is defined as:
            1. For patients who meet HA-VTE outcome criterion #2 (abnormal ultrasound) and/or #3 (#PE1) and/or #4 (#PE2): it is the time of the earliest of these three criteria
            2. For patients who do not meet HA-VTE criteria #2, #3, or #4 AND they meet criterion #1 (ICD codes): the time of HA-VTE would be defined as the earliest time in which any of the following HA-VTE criteria were met: #5 (AC clinic referral), #6 (AC clinic note/visit), #7 (medication for VTE), #8 (ICD for long-term AC), #9 (IVC filter), or #10 (*for this criterion #10, time would be timing of the specified relevant order sets and not time of death*)

        ii. Time to VTE from admission = Time of HA-VTE minus time of admission

© 2022 Neeman E et al. *JAMA Network Open.*

2. Time to first VTE from discharge: similar definition as “Time to VTE from admission” above but the calculation uses the time of discharge instead of time of HBS admission order set

© 2022 Neeman E et al. *JAMA Network Open.*

Definitions of HA-VTE predictors and risk factors

Padua Predictive Score components:

1. Active cancer: True if any of the following:
    a. ICD codes for cancer + ICD code for metastasis placed in the last 6 months prior to the index admission, and not present more than 6months prior to the admission. Do not include resolved problems from problem list
        i. ICD code for of “any malignancy”: ICD 9 codes: 140.x-172.x; 174.x-195.8; 200.x-208.x. ICD 10 codes: C00.x-C26.x; C30.x-C34.x; C37.x-C41.x; C43.x, C45.x-C58.x; C60.x-C76x; C81.x-C85.x; C88.x; C90.x-C97x; AND
        ii. ICD code of “metastatic solid tumor”: ICD 9 196.x-199.1; ICD 10 C77.x-C80.x

    OR

    b. Received any antineoplastic medication (chemotherapy, immunotherapy, targeted therapy, etc) in the 6 months prior to the index admission based on internal pharmacy codes. These included only completed orders for IV antineoplastic medications or filled outpatient prescriptions. Medications with non-oncological indications (e.g., rituximab) were excluded; OR
    c. The patient had any radiation oncology appointment/treatment in the 6 months prior to the index admission; OR
    d. The patient had new staging information in their problem list cancer staging module signed in the past 6 months with a stage III or IV cancer

© 2022 Neeman E et al. *JAMA Network Open.*

(including substages such as IIIa, IIIb, IVa, IVb, etc)

2. Previous VTE: true if at any point in time prior to day of index admission the patient had:
    a. ICD codes (including resolved problems from problem list):
        i. ICD 9: V12.51, V12.52, V12.55, 415.0, 415.11, 415.19, 416.2, 416.8, 444.1, 444.21, 444.22, 444.81, 444.89, 444.9, 451.11, 451.19, 451.81, 451.83, 453.2, 453.3, 453.40, 453.41, 453.42, 453.50, 453.51, 453.52, 453.72, 453.73, 453.74, 453.76, 453.77, 453.79, 453.82, 453.83, 453.84, 453.85, 453.86, 453.87, 453.89, 453.9, 673.20, 673.21, 673.22, 673.23, 673.24, 673.80, 673.81, 673.83, 673.84, 639.6.
        ii. ICD 10: I26.02, I26.09, I26.99, I27.24, I27.82, I74.11, I74.2, I74.3, I74.4, I74.5, I74.8, I74.9, I80.10, I80.11, I80.12, I80.13, I80.201, I80.202, I80.203, I80.209, I80.211, I80.212, I80.213, I80.219, I80.221, I80.222, I80.223, I80.229, I80.231, I80.232, I80.233, I80.239, I80.291, I80.292, I80.293, I80.299, I80.8, I82.210, I82.211, I82.220, I82.221, I82.290, I82.291, I82.3, I82.401, I82.402, I82.403, I82.409, I82.411, I82.412, I82.413, I82.419, I82.421, I82.422, I82.423, I82.429, I82.431, I82.432, I82.433, I82.439, I82.441, I82.442, I82.443, I82.449, I82.491, I82.492, I82.493, I82.499, I82.4Y1, I82.4Y2, I82.4Y3 ,I82.4Y9, I82.4Z1, I82.4Z2, I82.4Z3, I82.4Z9, I82.501, I82.502, I82.503, I82.509, I82.511, I82.512,

© 2022 Neeman E et al. *JAMA Network Open.*

I82.513, I82.519, I82.521, I82.522, I82.523, I82.529, I82.531, I82.532, I82.533, I82.539, I82.541, I82.542, I82.543, I82.549, I82.591, I82.592, I82.593, I82.599, I82.5Y1, I82.5Y2, I82.5Y3, I82.5Y9, I82.5Z1, I82.5Z2, I82.5Z3, I82.5Z9, I82.601, I82.602, I82.603, I82.609, I82.621, I82.622, I82.623, I82.629, I82.701, I82.702, I82.703, I82.709, I82.721, I82.722, I82.723, I82.729, I82.890, I82.891, I82.90, I82.91, I82.91, I82.A11, I82.A12, I82.A13, I82.A19, I82.A21, I82.A22, I82.A23, I82.A29, I82.B11, I82.B12, I82.B13, I82.B19, I82.B21, I82.B22, I82.B23, I82.B29, I82.C11, I82.C12, I82.C13, I82.C19, I82.C21, I82.C22, I82.C23, I82.C29, O08.2, O88.211, O88.211, O88.212, O88.212, O88.213, O88.213, O88.219, O88.22, O88.23, O88.811, O88.812, O88.813, O88.819, O88.82, O88.83, T81.718A, T81.72XA, T82.817A, T82.818A, Z86.711, Z86.718, Z86.72;

OR

b. Anticoagulation clinic visit progress note which included any of the following strings anywhere in the note: “VTE” OR “DVT” or “deep vein thrombosis” or “pulmonary embolism” but NOT any of following strings anywhere in the note: "prophylax" OR "PPX"

3. Reduced mobility/bedrest: True if any of the following:

© 2022 Neeman E et al. *JAMA Network Open.*

a. ICD9 codes (do not include resolved problems with these codes): 094.1, 344.00, 344.01, 344.02, 344.03, 344.04, 344.09, 344.1, 344.9 placed any time before index admission; OR
b. ICD10 codes (do not include resolved problems with these codes): G80.0, G80.1, G80.2, G80.8, G82.20, G82.21, G82.22, G82.50, I69.365, M62.3 placed any time before index admission; OR
c. “bedrest” or “bed rest” order placed during the encounter but prior to admission time (i.e., in the emergency room)
d. During the encounter and prior to admission time, true if nursing flowsheet variables CLOF (clinician assessed level of function) or PLOF (patient reported level of function) were indicative of severe dysfunction or bed bound

4. Thrombophilia: “true” if any of the following:
    a. Has ICD9 code: 289.81 OR any of these ICD10 codes: D68.5, D68.51, D68.52, D68.59, D68.6, D68.61, D68.62, D68.69 placed at any time before index admission (do not include resolved problems with these codes); OR
    b. Lab results obtained at any time prior to index admission identifying prothrombin gene mutation or factor V Leiden

5. Recent trauma/surgery: “true” if during the 30 days prior to the index admission an operating room was used for this patient (excluding all minor procedure suites

like cath-lab or GI suite)

6. Age 70 or above: “true” if age was ≥70 years at time of index admission

7. Heart or respiratory failure: any of the following:
   a. Heart failure: use of any dose of IV (not PO or any other form) of medication from pharmaceutical class loop diuretics during admission encounter prior to admission time. Internal pharmacy codes were used to identify these medications
   b. Respiratory failure: use of any oxygen supplementation higher than 4L/min at any point during index admission prior to admission time

8. Current admission for CVA or TIA: “true” if an order set for CVA or TIA management, or for thrombolytics was used during the admission encounter before admission time
   Note: In KPNC these order sets are mandated for any suspicion of TIA/CVA and are universally used.

   The original Padua Predictive Score element for stroke also included acute myocardial infarction, but we have excluded this variable as most patients with AMI would have received some therapeutic doses of anticoagulation early in their admission.

© 2022 Neeman E et al. *JAMA Network Open.*

9. Current admission for acute infection: true if any IV antibiotics given (order was completed) during current admission before time of admission. Internal pharmacy codes were used to identify these medications

   Notably, the original Padua Score elements combined acute infection with acute rheumatological disorders, but we have excluded the latter since in our system these are almost exclusively treated in the outpatient setting.

10. BMI≥30 *(weight[kg]/(height[m])^2)*: Calculate BMI on day of index admission (weight in kg on day of index admission, or if not available most recent weight in kg prior to index admission, divided by squared most recent height in meters on or prior to day of index admission). True if BMI=30 or higher. Note: we found rare instances when weight or height were entered incorrectly, thus we excluded this variable (left as "missing" if BMI≤12 or ≥60)

11. Ongoing hormonal treatment: true if of the criteria below is positive:
    a. if has an active ICD code in problem list for hormonal treatment on day of index admission:
       ICD-10: Z79.890, Z79.81 or ICD-9: V07.4
    b. any relevant medications (administered in office or dispensed at outpatient pharmacy) up to 6 months prior to index admission. Internal pharmacy codes were used to identify these medications

© 2022 Neeman E et al. *JAMA Network Open.*

Additional identified risk factors

12. Active tobacco smoker: True if at time of index admission, the Social History documentation in the EHR was one of the following:
    a. Current Every Day Smoker; OR
    b. Current Some Day Smoker; OR
    c. Smoker, Current Status Unknown; OR
    d. Heavy Tobacco Smoker; OR
    e. Light Tobacco Smoker

13. Poorly controlled diabetes: True if the last hemoglobin-A1c test was resulted within a year prior to time of admission time and was = 10.0% or higher

14. Low hemoglobin: true if the last hemoglobin test was resulted within a month prior to time of admission and was lower than 10.0

15. High platelet count: true if: True if the most recent platelet test was resulted within a month prior to tome of admission and was = 500,000 or higher

16. PICC line or infusion port: true if at the time of admission the patient had any type of line which included the word “PICC” or “port” (under LDA or “lines/drains/airways” in nursing flowsheets)

© 2022 Neeman E et al. *JAMA Network Open.*

17. Use of serotonergic antidepressants: true if a prescription of any medication from the following pharmaceutical subclasses was dispensed in the pharmacy up to 6 months prior to index admission: Serotonin-Norepinephrine Reuptake Inhibitors (SNRIs), Selective Serotonin Reuptake Inhibitors (SSRIs), Menopausal Symptoms Suppressant-SSRI Antidepressant Type, Serotonin-2 Antagonist-Reuptake Inhibitors (SARIs). Internal pharmacy codes were used to identify these medications

18. Microalbuminuria: true if in within 6 months prior to admission date the patient had any abnormal result of the urine albumin/creatinine ratio lab test

19. COmorbidity Point Score ≥50 (COPS; with one year lookback period) and Laboratory Acute Physiology score ≥ 100 (LAPS; with 72-hour lookback) on day of admission. COPS and LAPS are illness severity indices that can be automatically calculated in real time, and were developed and validated at KPNC[1].

20. Recent major hemorrhage: if any of the following ICD codes were entered in encounter diagnoses or problem list within 30 days prior to date of admission:
    a. ICD 9: 423, 423.1, 430, 431, 432.1, 432.9, 455.2, 455.5, 455.8, 456, 456.1, 456.2, 456.21, 456.8, 459, 459.31, 459.32, 459.33, 459.81, 530.7, 531, 531.1, 531.11, 531.21, 531.3,531.4, 531.41, 531.5, 531.51, 531.61, 532, 532.1, 532.11, 532.3, 532.4, 532.5, 532.6, 533, 533.3, 533.4, 533.5,

© 2022 Neeman E et al. *JAMA Network Open.*

534.1, 534.4, 534.5, 534.6, 5350.1, 535.11, 535.31, 535.4, 535.41, 535.51, 535.6, 535.61, 537.83, 562.12, 562.13, 568.81, 569.3, 569.85, 578, 578.1, 578.9, 719.11, 784.7, 784.8, 786.3, 852, 852.01, 852.06, 852.2, 852.21, 852.26, 852.29, 852.4, 852.41, 852.46, 853, 853.01, 853.06, 853.09

b. ICD 10: I31.2, I60.9, I61.9, I62.1 I62.9, I85.01, I85.11, K22.6, K22.8, K25.0, K25.1, K25.2, K25.3, K25.4, K25.5, K25.6, K26.0, K26.1, K26.2, K26.3, K26.4, K26.5, K26.6, K27.0, K27.1, K27.2, K27.3, K27.4, K27.5, K27.6, K28.1, K28.3, K28.4, K28.5, K29.01, K29.21, K29.41, K29.51, K29.61,K29.71, K29.91, K31.811, K55.21, K57.11, K57.13, K57.31, K57.33, K62.5, K62.5, K64.4, K64.8, K66.1, K92.0, K92.1, K92.2, R04.0, R04.2, R04.89, R04.9, R31.0, R31.1, R31.21, R31.29, R31.9, R58, S06.300A, S06.300D, S06.300S, S06.301A, S06.301S, S06.307S, S06.308A, S06.308S, S06.309A, S06.309D, S06.309S, S06.310A, S06.319D, S06.320A, S06.321A, S06.329A, S06.329D, S06.329S, S06.330A, S06.331A, S06.331D, S06.339A, S06.339D, S06.339S, S06.340A, S06.340D, S06.341A, S06.341D, S06.341S, S06.349A, S06.349D, S06.349S, S06.359A, S06.359S, S06.360A, S06.360S, S06.361A, S06.362S, S06.369A, S06.369D, S06.369S, S06.370A, S06.370D, S06.370S, S06.372A, S06.379A, S06.379D, S06.379S, S06.380A, S06.389A, S06.4X0A, S06.4X0D, S06.4X0S, S06.4X1A, S06.4X1D, S06.4X2A, S06.4X9A, S06.4X9D, S06.4X9S, S06.5X0A, S06.5X0D, S06.5X0S, S06.5X1A, S06.5X1D, S06.5X1S, S06.5X2D,

S06.5X2S, S06.5X4A, S06.5X4D, S06.5X5D, S06.5X5S, S06.5X6A, S06.5X9A, S06.5X9A, S06.5X9D, S06.5X9S, S06.6X0A, S06.6X0D, S06.6X0S, S06.6X1A, S06.6X1D, S06.6X1S, S06.6X2D, S06.6X5D, S06.6X9A, S06.6X9D, S06.6X9S

21. Race and ethnicity: as documented in the EHR at time of admission. At KPNC race and ethnicity are self-reported as two separate variables.

22. Requirement for language interpreter: as documented in the EHR at time of admission. If no value was entered, this was considered to be “no requirement”

© 2022 Neeman E et al. *JAMA Network Open.*

Definitions of HA-VTE prophylaxis

- Theoretical definition: Did the patient receive any dose of pharmacological prophylaxis during the first 48h of admission
- Operational Definition: Binary outcome. "True" if one of the medications below was given (order for the medication was completed) during the first 48 hours since time of admission:
    - Enoxaparin if given once daily with dose being <0.8mg/kg (where kg is based on the most recent weight prior to the enoxaparin order) OR if given at 30mg twice a day regardless of weight
    - Heparin if given at dose of 5000-7500 Units subcutaneous (not intravenous) at any interval (once, twice or more per day)
    - Note: no other agents (e.g., fondaparinux, dalteparin) are routinely used for HA-VTE prophylaxis in our healthcare system. Internal pharmacy codes were used to identify these medications above

Definitions of therapeutic anticoagulation in first 48 hours

- Note: this definition was used as part of the exclusion criteria (see Methods section)
- Theoretical definition: received order for therapeutic anticoagulation <48 hrs after time of admission
- Operational Definition: "true" if an order for one of the drugs below is signed within 48 hours after initiation of index admission. The order has to be completed (not cancelled) at any time point, but signed within 48h as above
    - Warfarin any dose/regimen

© 2022 Neeman E et al. *JAMA Network Open.*

- Apixaban any dose/regimen
- Rivaroxaban any dose/regimen
- Dabigatran any dose/regimen
- Enoxaparin if given twice a day at a dose > 30mg or three times a day at any dose, or if given once daily with dose being >=0.8mg/kg where kg is based on the most recent weight prior to the enoxaparin order
- Heparin: true if given intravenously (not subcutaneous) at any dose >1000 units in total (*for example, 100 units/ml given 5ml total =500 would not be included*)
- Note: Internal pharmacy codes were used to identify these medications above

© 2022 Neeman E et al. *JAMA Network Open.*
